# Accurate and noninvasive prostate cancer detection using plasma‐derived extracellular vesicle RNA

**DOI:** 10.1002/2211-5463.70305

**Published:** 2026-08-07

**Authors:** Hanping Wei, Haoran Wu, Wei Feng

**Affiliations:** ^1^ Department of Urology Wujin Hospital Affiliated with Jiangsu University Changzhou China; ^2^ Department of Urology The Wujin Clinical College of Xuzhou Medical University Changzhou China

**Keywords:** extracellular vesicles, machine learning analysis, plasma, Prostate cancer, RNA, RT–qPCR, WGCNA

## Abstract

Prostate cancer (PCa) is the most commonly diagnosed noncutaneous malignancy in men and a leading cause of cancer‐related death worldwide. Its clinical heterogeneity, ranging from indolent to aggressive disease, presents major diagnostic and therapeutic challenges. However, prostate‐specific antigen (PSA), the current standard biomarker, lacks sufficient sensitivity and specificity, resulting in both overdiagnosis and missed cases. Therefore, more accurate noninvasive biomarkers are urgently needed. In this study, we isolated plasma‐derived extracellular vesicles (EVs) using a wheat germ agglutinin (WGA)‐conjugated magnetic bead method and identified differentially expressed transcripts between PCa patient and healthy control (HC) EVs. Candidate biomarkers were selected through weighted gene co‐expression network analysis (WGCNA) and Random Forest modeling, followed by validation using RT‐qPCR in independent cohorts including PCa, benign prostatic hyperplasia (BPH), and HC samples. We identified three EV‐derived RNAs (NM_024955, NR_047469, and NR_002564), which were significantly dysregulated in PCa compared with both HC and BPH. Combined analysis demonstrated high diagnostic performance, outperforming PSA and notably NM_024955 expression showed a strong correlation, indicating potential relevance to disease severity. These findings suggest that lectin‐based EV isolation coupled with RNA profiling provides a robust and scalable platform for PCa diagnosis, offering promising noninvasive biomarkers to complement or improve current PSA‐based screening.

## Graphical abstract

Plasma extracellular vesicles were captured with WGA‐conjugated magnetic beads and profiled for RNA biomarkers. A three‐RNA panel (NM_024955, NR_047469, and NR_002564) distinguished prostate cancer from healthy controls and benign prostatic hyperplasia, supporting a simple, noninvasive approach to improve prostate cancer detection. License statement: The graphical abstract was created using original vector‐style drawings; BioRender or a similar service was not used*.*

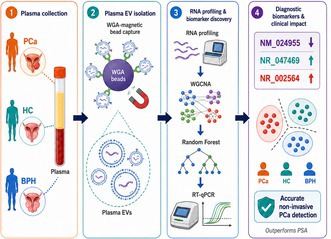

**Abbreviations**
AD: Alzheimer's disease
AUC: area under the curve
BPH: benign prostatic hyperplasia
BPH: benign prostatic hyperplasia
cDNA: Complementary DNA
circRNAs: circular RNAs
ER: endoplasmic reticulum
ERAD: endoplasmic reticulum‐associated degradation
EV: extracellular vesicle
FDR: false discovery rate
FOXRED2: FAD‐dependent oxidoreductase domain‐containing protein 2 (FOXRED2)
GlcNAc: N‐acetyl‐D‐glucosamine
GO: Gene Ontology
GS: Gleason score
HC: healthy control
KEGG: Kyoto Encyclopedia of Genes and Genomes
lncRNAs: long non‐coding RNAs
miRNAs: microRNAs
mRNAs: messenger RNAs
NTA: Nanoparticle Tracking Analysis
PBS: phosphate‐buffered saline
PCA: principal component analysis
PCa: Prostate cancer
PSA: prostate‐specific antigen
RF: Random Forest
ROC: receiver operating characteristic
RT‐qPCR: Reverse Transcription Quantitative Polymerase Chain Reaction
SD: standard deviation
TEM: Transmission Electron Microscopy
TOM: Topological Overlap Matrix
WGA: wheat germ agglutinin
WGCNA: weighted gene co‐expression network analysis

Prostate cancer (PCa) ranks as the most common nonskin malignancy diagnosed in men globally and is a major contributor to cancer‐related deaths in the male population [1]. The disease exhibits a wide clinical spectrum, ranging from slow‐growing, localized tumors with minimal progression risk to aggressive forms capable of metastasis [2]. This variability creates significant challenges for clinicians, as excessive treatment of low‐risk cases and insufficient treatment of high‐risk disease can both negatively impact patient survival and quality of life [2]. The long‐standing biomarker used for PCa detection and follow‐up, prostate‐specific antigen (PSA), suffers from well‐documented shortcomings in both sensitivity and specificity [3]. PSA elevation is not exclusive to cancer; it may also occur in benign prostatic hyperplasia or prostatitis, leading to unnecessary biopsy procedures and increased patient distress [4]. On the other hand, some patients with clinically significant PCa may present with PSA values within the normal range, resulting in delayed or missed diagnoses [5]. Therefore, the search for more reliable biomarkers is crucial for earlier disease identification, improved risk assessment, prediction of therapeutic response, and ongoing monitoring of tumor progression [2, 6].

Extracellular vesicles, small extracellular vesicles generally about 100 nm in size, though measurements can extend up to 150–200 nm depending on the source cell type and isolation technique, are gaining recognition as highly promising biomarker carriers in PCa research [7, 8]. Originating from multivesicular bodies, these vesicles are loaded with diverse nucleic acids, including microRNAs (miRNAs), long noncoding RNAs (lncRNAs), messenger RNAs (mRNAs), and circular RNAs (circRNAs) [7, 8]. Accumulating evidence suggests that extracellular vesicle RNAs play important roles in prostate tumor development and progression by regulating essential signaling and molecular pathways [9]. Nonetheless, clinical application of RNA biomarkers within extracellular vesicles is hindered by the lack of straightforward, efficient, and scalable isolation and profiling methods [10]. Conventional approaches tend to be labor‐intensive, time‐consuming, and poorly suited for routine diagnostics. To overcome these obstacles, researchers have introduced a lectin‐conjugated magnetic bead method for extracellular vesicle isolation, providing a more practical and scalable option than many existing protocols [11, 12, 13, 14].

In this study, we employed the lectin‐based isolation technique on plasma‐derived extracellular vesicles, integrating it with RT‐qPCR to measure RNA expression levels. Through this approach, we identified NM_024955, NR_047469, and NR_002564 as potential extracellular vesicle‐based plasma biomarkers for diagnosing PCa. Our results indicate that this strategy could enable noninvasive and potentially earlier disease detection, while also enhancing patient monitoring and informing clinical decision‐making.

## Materials and methods

### Experimental design

To identify plasma extracellular vesicle RNA biomarkers for noninvasive diagnosis of prostate cancer (PCa), this study was conducted in two sequential phases: a discovery phase based on RNA sequencing and bioinformatic screening, followed by an independent validation phase using RT‐qPCR. Plasma extracellular vesicles were isolated from PCa patients and healthy controls (HCs) using WGA‐conjugated magnetic beads, as described previously [11, 12, 13, 14, 15, 16]. Total extracellular vesicle RNA was extracted and subjected to high‐throughput RNA sequencing. After quality control and expression normalization, differentially expressed RNAs between PCa and HC samples were identified.

To prioritize candidate diagnostic biomarkers, we integrated three complementary analytical approaches. First, Weighted Gene Co‐expression Network Analysis (WGCNA) [17] was performed to construct co‐expression modules and identify RNA modules significantly associated with PCa status. A soft‐thresholding power was selected to approximate scale‐free topology, and module–trait relationships were evaluated to determine disease‐related modules. Second, a Random Forest machine learning model [18] was used to further rank the diagnostic importance of candidate RNAs. The input features included dysregulated RNAs identified from sequencing data, and the disease status of each sample was used as the classification label. The Random Forest model was trained to distinguish PCa from HC samples, and RNA importance was ranked according to the mean decrease in classification accuracy and/or Gini impurity. The top‐ranked RNAs from the Random Forest analysis were considered machine‐learning‐supported diagnostic candidates. Third, candidate RNAs were selected by intersecting the differentially expressed RNAs, PCa‐associated WGCNA module genes, and the top Random Forest‐ranked features.

RNAs shared by these three analyses were considered robust biomarker candidates and were subsequently validated by RT‐qPCR in an independent clinical cohort comprising PCa patients, HCs, and benign prostatic hyperplasia (BPH) cases. The diagnostic performance of individual RNAs and their combined panel was assessed using receiver operating characteristic (ROC) curve analysis. Sensitivity, specificity, and area under the curve (AUC) values were calculated to evaluate their ability to distinguish PCa from HC and BPH samples. The diagnostic performance of the RNA panel was further compared with serum PSA.

### Clinical samples

A total of 313 participants were enrolled from Wujin Hospital Affiliated with Jiangsu University, including 158 patients with pathologically confirmed prostate cancer (PCa), 104 healthy controls (HCs), and 51 patients diagnosed with benign prostatic hyperplasia (BPH). All PCa cases were classified as stage I at the time of sample collection. To ensure methodological clarity, the study cohort was divided into two independent sets. A subset of samples (38 PCa patients and 54 HCs) was randomly selected for the RNA sequencing‐based discovery phase. Clinical and demographic characteristics of all participants are summarized in Table S1. The remaining samples (120 PCa patients, 50 HCs, and 51 BPH patients) were used as an independent validation cohort for RT‐qPCR analysis. Clinical and demographic characteristics of all participants are summarized in Table S2. All participants were recruited under protocols approved by the Institutional Review Board of the Human Subjects Research Ethics Committee of Wujin Hospital (approval number: 2024‐SR), and the study was conducted in accordance with the Declaration of Helsinki. Written informed consent was obtained from all individuals prior to inclusion. Exclusion criteria included the presence of other malignancies, severe systemic diseases, or any condition that could potentially influence circulating extracellular vesicle profiles.

### Extracellular vesicles isolation using WGA‐conjugated magnetic beads

Plasma extracellular vesicles (EVs) were isolated using WGA‐conjugated magnetic beads, as previously described [11, 12, 13, 14, 15, 16]. (1) EV isolation for characterization (Western blotting, nanoparticle tracking analysis, and transmission electron microscopy), 1 mL of plasma was centrifuged at 3000 × g for 10 min at 4°C to remove cellular debris, followed by filtration through a 0.45 μm membrane (Millipore, HAWP04700). The clarified plasma was then incubated with WGA‐conjugated magnetic beads at room temperature for 1 h with gentle rotation. After incubation, samples were placed on a magnetic rack for 5 min to collect bead‐bound EVs. The supernatant was discarded, and the beads were washed twice with phosphate‐buffered saline (PBS). EVs were subsequently eluted using a buffer containing 500 mm N‐acetyl‐D‐glucosamine (GlcNAc). The elution procedure consisted of: (i) washing 20 μL of WGA beads three times with 400 μL PBS; (ii) adding 400 μL of GlcNAc elution buffer per 20 μL of beads and incubating at room temperature for 15 min with rotation; (iii) magnetic separation of beads; and (iv) collection of the eluate for downstream western blotting, NTA, and TEM analyses. (2) EV isolation for RNA sequencing and RT‐qPCR, 1 mL of plasma was processed as described above, including centrifugation at 3000 × g for 10 min and filtration through a 0.45 μm membrane. The supernatant was incubated with WGA‐conjugated magnetic beads at room temperature for 1 h. Following magnetic separation, the supernatant was removed and the beads were washed twice with PBS to reduce potential contaminants. Total RNA was directly extracted from EV‐bound beads by adding 1 mL of TRIzol reagent (Invitrogen, USA) according to the manufacturer's instructions. The extracted RNA was subsequently used for RNA sequencing and RT‐qPCR analyses.

### Nanoparticle tracking analysis (NTA)

Particle size distribution and concentration of EVs were determined using the NanoSight NS300 system (Malvern, UK), equipped with a 488‐nm laser and a high‐sensitivity scientific CMOS (sCMOS) camera. The sample chamber temperature was maintained at 25 C during analysis. Each sample was measured under the following parameters: camera level set to 12, acquisition time of 30 s, and detection threshold of 7. Three independent recordings were captured for each sample to ensure reproducibility. The obtained data were analyzed using the NanoSight software (version 2.3).

### Transmission electron microscopy (TEM)

The morphology and ultrastructure of EVs were examined by TEM. EV samples were fixed with 2.5% glutaraldehyde and 5% bovine serum albumin (BSA), followed by postfixation with 2% osmium tetroxide. Samples were dehydrated through a graded series of acetone and ethanol and subsequently embedded in epoxy resin (SPI Inc., West Chester, PA, USA). Ultrathin sections (80–90 nm) were prepared and stained sequentially with 5% uranyl acetate for 15 min and 0.1% lead citrate for 5 min. Images were captured using a Hitachi 7500 transmission electron microscope.

### Western blot analysis

EV proteins were extracted using RIPA lysis buffer (P0013B, Beyotime) supplemented with phenylmethylsulfonyl fluoride (PMSF; ST506, Beyotime) and a protease inhibitor cocktail (P1046, Beyotime). Equal amounts of protein were separated by sodium dodecyl sulfate‐polyacrylamide gel electrophoresis and transferred onto polyvinylidene fluoride membranes (Millipore, USA). Membranes were blocked with 5% skim milk for 1 h at room temperature and incubated overnight at 4 °C with primary antibodies (dilution 1:1000), including CD63 (52090S, CST), ALIX (12422‐1‐AP, Proteintech), and Calnexin (10427‐2‐AP, Proteintech). After washing four times with Tris‐buffered saline containing 0.1% Tween‐20, membranes were incubated with horseradish peroxidase‐conjugated secondary antibodies (ab205718, Abcam) for 1 h at room temperature. Protein bands were visualized using enhanced chemiluminescence reagents (Millipore, USA).

### RNA extraction from extracellular vesicles

Total RNA was extracted from extracellular vesicles using TRIzol reagent (Invitrogen, USA) according to the manufacturer's instructions with minor modifications. Briefly, EV pellets were lysed in TRIzol reagent and incubated at room temperature for 10 min to ensure complete dissociation of nucleoprotein complexes. Subsequently, 200 μL of chloroform was added, and the samples were vigorously mixed and incubated at room temperature for an additional 10 min. The mixture was then centrifuged at 12000 × g for 30 min at 4 °C to achieve phase separation. The aqueous phase containing RNA was carefully transferred to a new tube and mixed with an equal volume of isopropanol. To enhance RNA precipitation, samples were incubated at −20 °C for 1 h, followed by centrifugation at 12000 × g for 20 min at 4 °C. The resulting RNA pellet was washed with 75% ethanol, air‐dried, and resuspended in 10 μL of nuclease‐free water. RNA concentration and purity were assessed using a OneDrop‐2000 spectrophotometer (NanoDrop Technologies).

### RNA sequencing and bioinformatic processing

Total extracellular vesicle RNA was extracted from 5 mL of plasma using 50 μL of WGA‐conjugated magnetic beads, followed by TRIzol reagent (Invitrogen, USA) extraction as described above. RNA concentration and integrity were assessed using a NanoDrop 8000 UV–Vis spectrophotometer (Thermo Fisher Scientific, USA) and an Agilent 4200 TapeStation system (Agilent Technologies, USA). RNA sequencing libraries were constructed using the TruSeq RNA Access Library Prep Kit (Illumina, USA) according to the manufacturer's instructions. Sequencing was performed on an Illumina HiSeq 2500 platform in 100‐bp paired‐end mode using TruSeq Rapid PE Cluster and SBS kits. Raw sequencing reads were first subjected to quality control to remove low‐quality reads and adapter sequences. Clean reads were then aligned to the human reference genome (hg19) using the STAR aligner. Gene expression levels were quantified using RNA‐Seq by Expectation–Maximization and normalized as transcripts per million for downstream analyses. Quality control metrics, including mapping rate and transcript coverage, were evaluated using RNA‐SeQC. For exploratory analysis, principal component analysis (PCA) and differential expression analysis were performed to identify RNAs with significant expression differences between PCa and HC samples. Gene fusion events were detected using multiple algorithms, including ChimeraScan, deFuse, FusionMap, MapSplice, and TopHat, to improve detection robustness. The RNA sequencing cohort consisted of 38 patients with stage I PCa and 54 healthy controls. Detailed clinical and demographic characteristics of these subjects are provided in Table S1.

### Weighted gene Co‐expression network analysis (WGCNA)

Weighted Gene Co‐expression Network Analysis (WGCNA) was performed using the R package WGCNA [17] to identify co‐expression modules of extracellular vesicle RNAs associated with prostate cancer (PCa). Gene expression data obtained from RNA sequencing were used as input after normalization. Pairwise Pearson's correlation coefficients were calculated between all RNA expression profiles across 58 samples (38 PCa and 54 HCs). An appropriate soft‐thresholding power (β) was selected based on the criterion of approximate scale‐free topology (scale‐free topology fit index *R* ^2^ > 0.85). The resulting correlation matrix was transformed into an adjacency matrix and further converted into a Topological Overlap Matrix (TOM) to measure network connectivity. Hierarchical clustering based on TOM dissimilarity was performed to group RNAs with similar expression patterns. Gene modules were identified using the dynamic tree cut algorithm with a minimum module size of 30 genes, and modules with high similarity were merged using a merge cut height of 0.25. Each module was assigned a unique color identifier. To evaluate the association between modules and clinical traits, module eigengenes (the first principal component of each module) were calculated and correlated with disease status (PCa vs. HC) using Pearson's correlation analysis. Modules showing the strongest correlation with PCa were considered key disease‐related modules and were selected for subsequent biomarker screening and integration with machine learning analysis.

### Biomarker selection and machine learning analysis

Differential expression analysis of extracellular vesicle RNAs between PCa patients and healthy controls was performed using the limma R package. RNAs with |log_2_ fold change (FC)| > 1 and adjusted *P*‐value (false discovery rate, FDR) < 0.05 were considered significantly differentially expressed. To further identify robust diagnostic biomarkers, a Random Forest (RF) model was employed for feature selection using the randomForest R package. The input features consisted of the differentially expressed RNAs identified by limma analysis. The RF model was trained to classify PCa and HC samples using default parameters with 500 trees (ntree = 500). Feature importance was evaluated based on the mean decrease in Gini impurity, and RNAs were ranked accordingly. The top‐ranked features were selected as candidate biomarkers. To enhance the robustness of biomarker selection, results from three complementary approaches‐differential expression analysis (limma), Weighted Gene Co‐expression Network Analysis (WGCNA), and Random Forest feature selection‐were integrated. Specifically, RNAs that were simultaneously identified as (i) significantly differentially expressed, (ii) belonging to PCa‐associated WGCNA modules, and (iii) top‐ranked features in the Random Forest model were considered key candidate biomarkers. The overlap among these three methods was visualized using a Venn diagram, and the intersecting RNAs were selected for subsequent experimental validation.

### Reverse transcription quantitative PCR (RT‐qPCR)

Extracellular vesicles were isolated from 100 μL of plasma diluted with 900 μL phosphate‐buffered saline (PBS) using 10 μL of WGA‐conjugated magnetic beads. Total RNA was extracted from EVs using TRIzol reagent (Invitrogen, USA) according to the manufacturer's instructions. RNA concentration and purity were assessed using a OneDrop‐2000 spectrophotometer (NanoDrop Technologies). Complementary DNA (cDNA) was synthesized using the 1st Strand cDNA Synthesis Kit (Vazyme Biotech, China). Briefly, 8 μL of total RNA was mixed with 2 μL of 5 × gDNA Wiper Mix and incubated at 42 °C for 2 min to remove genomic DNA contamination. Subsequently, reverse transcription was performed by adding 2 μL of 10 × RT Mix, 2 μL of HiScript III Enzyme Mix, 1 μL of random hexamer primers, and 5 μL of RNase‐free water, followed by incubation at 37 °C for 45 min. Quantitative PCR was carried out in 96‐well plates using ChamQ Universal SYBR qPCR Master Mix (Vazyme Biotech, China). Each reaction contained 10 μL of 2 × SYBR Master Mix, 1 μL of gene‐specific primer mix, and the cDNA template in a final reaction volume according to the manufacturer's protocol. The amplification conditions were as follows: initial denaturation at 95 °C for 30 s, followed by 40 cycles of 95 °C for 10 s and 60 °C for 30 s. U6 small nuclear RNA (snRNA) was used as the internal control for normalization. Relative RNA expression levels were calculated using the 2^−ΔΔCt^ method. All reactions were performed in triplicate to ensure reproducibility. The validation cohort included 120 patients with stage I PCa, 50 healthy controls, and 51 BPH patients. U6 small nuclear RNA (snRNA) was used as the internal reference for normalization. The selection of U6 was based on its relatively stable expression across samples in this study. Detailed clinical information is provided in Table S2, and primer sequences are listed in Table S3.

### Statistical analysis

All statistical analyses were performed using GraphPad Prism 8.0 (GraphPad Software, USA) and R software (version 4.2.0). Data are presented as mean ± standard deviation (SD) unless otherwise indicated. For comparisons between two groups, an unpaired two‐tailed Student's *t*‐test was used. For comparisons among three or more groups, one‐way analysis of variance (ANOVA) followed by Tukey's *post hoc* test was applied. The normality of data distribution was assessed prior to parametric testing, and nonparametric tests were used when appropriate. Receiver operating characteristic (ROC) curve analysis was performed to evaluate the diagnostic performance of candidate biomarkers, and the area under the curve (AUC), sensitivity, and specificity were calculated. Correlation analysis was conducted using Pearson's correlation coefficients. A *P*‐value < 0.05 was considered statistically significant. Where applicable, multiple testing correction was performed using the Benjamini–Hochberg method to control the false discovery rate (FDR). Given the imbalance in sample sizes between PCa and control groups, diagnostic performance was primarily evaluated using receiver operating characteristic (ROC) curve analysis, which is less sensitive to class imbalance.

## Results

### Characterization of extracellular vesicles isolated via WGA‐coupled magnetic beads

**Fig. 1:**
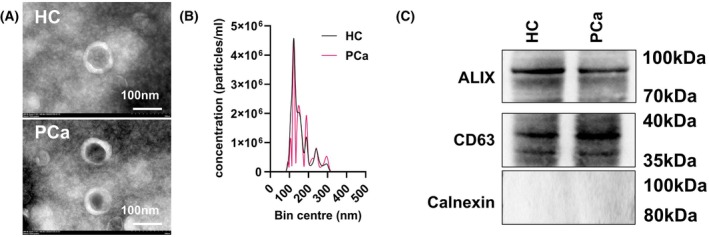
Isolation and characterization of plasma extracellular vesicles. (A) TEM analysis of plasma extracellular vesicles. The scale bar represents 100 nm. (B) NTA quantification of plasma extracellular vesicles. (C) Western blotting analysis of plasma extracellular vesicles. PCa: Prostate cancer; HC: healthy control.

Extracellular vesicles were successfully isolated from plasma samples using WGA‐conjugated magnetic beads. The morphology, size distribution, and protein marker expression of the isolated EVs were characterized by transmission electron microscopy (TEM), nanoparticle tracking analysis (NTA), and western blotting. TEM analysis revealed that the isolated EVs exhibited a typical cup‐shaped morphology with a bilayer membrane structure (Fig. 1A). NTA showed that the EVs had a size distribution centered around approximately 100 nm in diameter (Fig. 1B), consistent with the expected size range of extracellular vesicles. Western blot analysis further confirmed the identity of the isolated EVs. Established EV markers, including CD63 and ALIX, were clearly detected, whereas Calnexin, an endoplasmic reticulum protein commonly used as a negative control, was not detected in the EV samples (Fig. 1C and Fig. S1). These results indicate that the isolated vesicles were of high purity and consistent with the characteristics of extracellular vesicles.

### Expression profile of RNAs in plasma extracellular vesicles from PCa patients and HCs

**Fig. 2:**
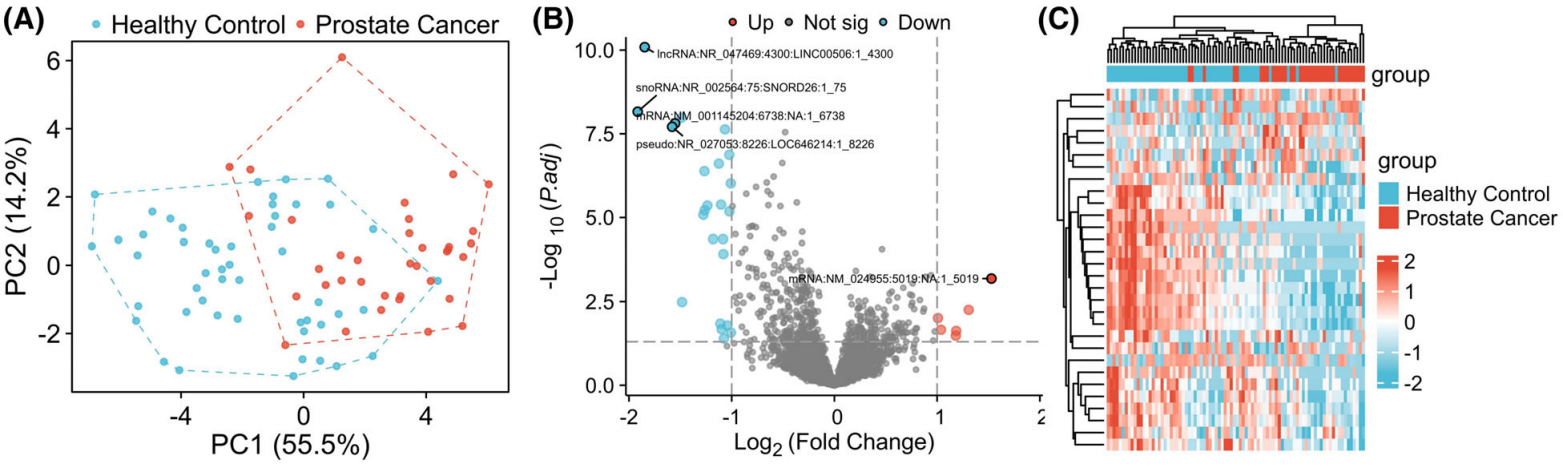
Principal component analysis (A), volcano plot (B), and heatmap (C) analysis of RNA sequencing data of plasma extracellular vesicles from prostate cancer patients and healthy controls.

RNA sequencing analysis revealed distinct transcriptomic differences in plasma‐derived extracellular vesicles between prostate cancer (PCa) patients and healthy controls (HCs). Principal component analysis (PCA) demonstrated a clear separation between the two groups, indicating substantial differences in global RNA expression profiles (Fig. 2A). Differential expression analysis identified a total of 27 RNAs with significant expression changes in PCa‐derived EVs compared with HCs, including six upregulated and 21 downregulated transcripts (Fig. 2B, Table S4). Consistently, hierarchical clustering analysis further demonstrated that these differentially expressed RNAs could effectively distinguish PCa samples from HCs, as shown by the distinct clustering patterns in the heatmap (Fig. 2C).

Taken together, these results indicate that plasma EV‐derived RNAs exhibit disease‐specific expression patterns and may serve as potential biomarkers for PCa.

### Functional enrichment analysis of dysregulated RNAs in extracellular vesicles

**Fig. 3:**
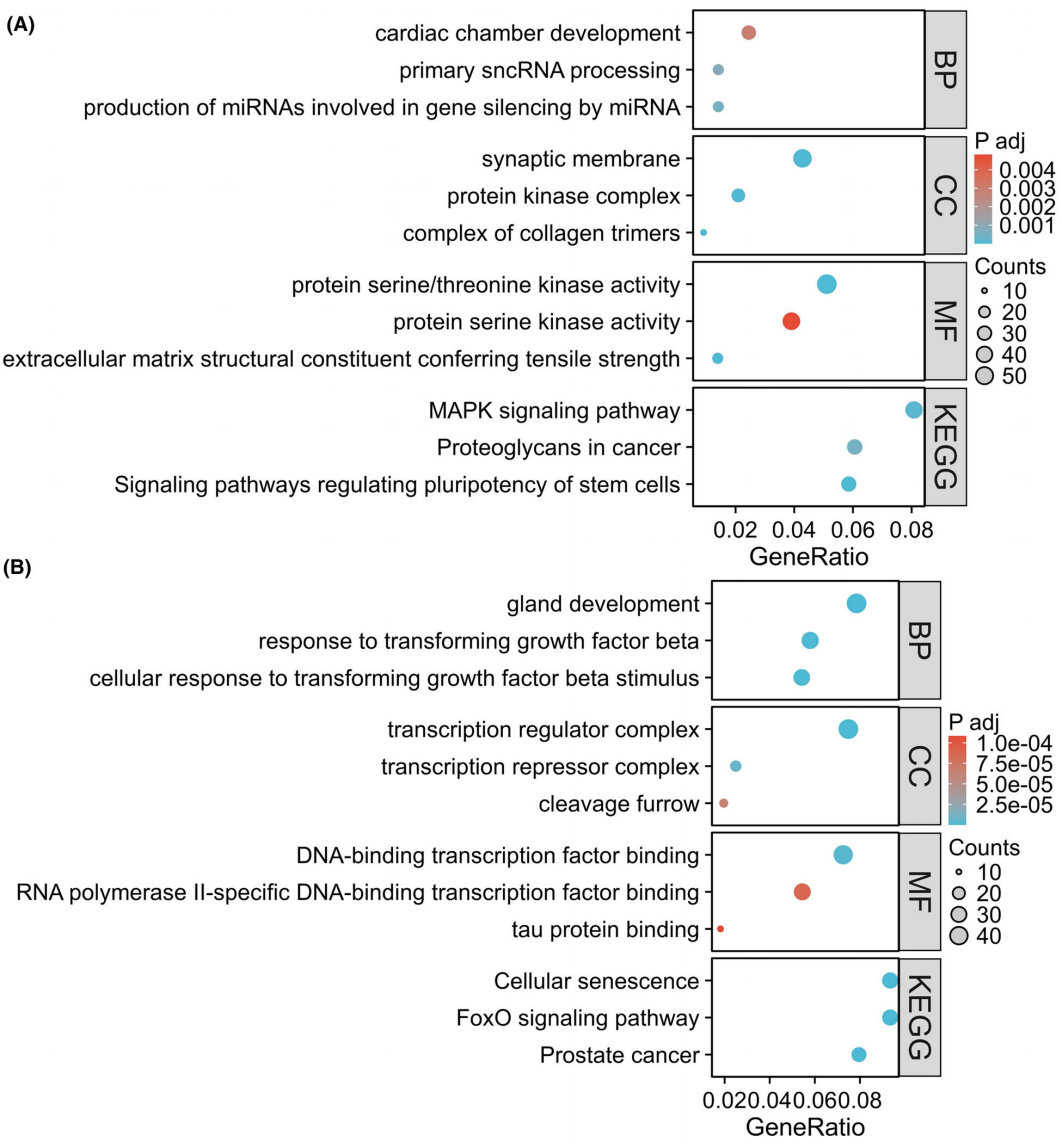
GO and KEGG analyses of upregulated (A) and downregulated (B) RNAs. GO: Gene Ontology; KEGG: Kyoto Encyclopedia of Genes and Genomes; BP: Biological Process; CC: Cellular Component; MF: Molecular Function.

To explore the biological relevance of the dysregulated RNAs identified in plasma extracellular vesicles (EVs), Gene Ontology (GO) and Kyoto Encyclopedia of Genes and Genomes (KEGG) pathway enrichment analyses were performed [19, 20, 21]. Upregulated RNAs were significantly enriched in pathways associated with the MAPK signaling cascade, proteoglycans in cancer, and the regulation of stem cell pluripotency (Fig. 3A, Table S5). In contrast, downregulated RNAs were primarily enriched in FoxO signaling, prostate cancer‐related pathways, and cellular senescence processes (Fig. 3B, Table S6). These enrichment results suggest that EV‐derived RNAs are involved in key cancer‐related signaling pathways and may contribute to the development and progression of prostate cancer.

### Identification of candidate biomarkers by WGCNA and machine learning

**Fig. 4:**
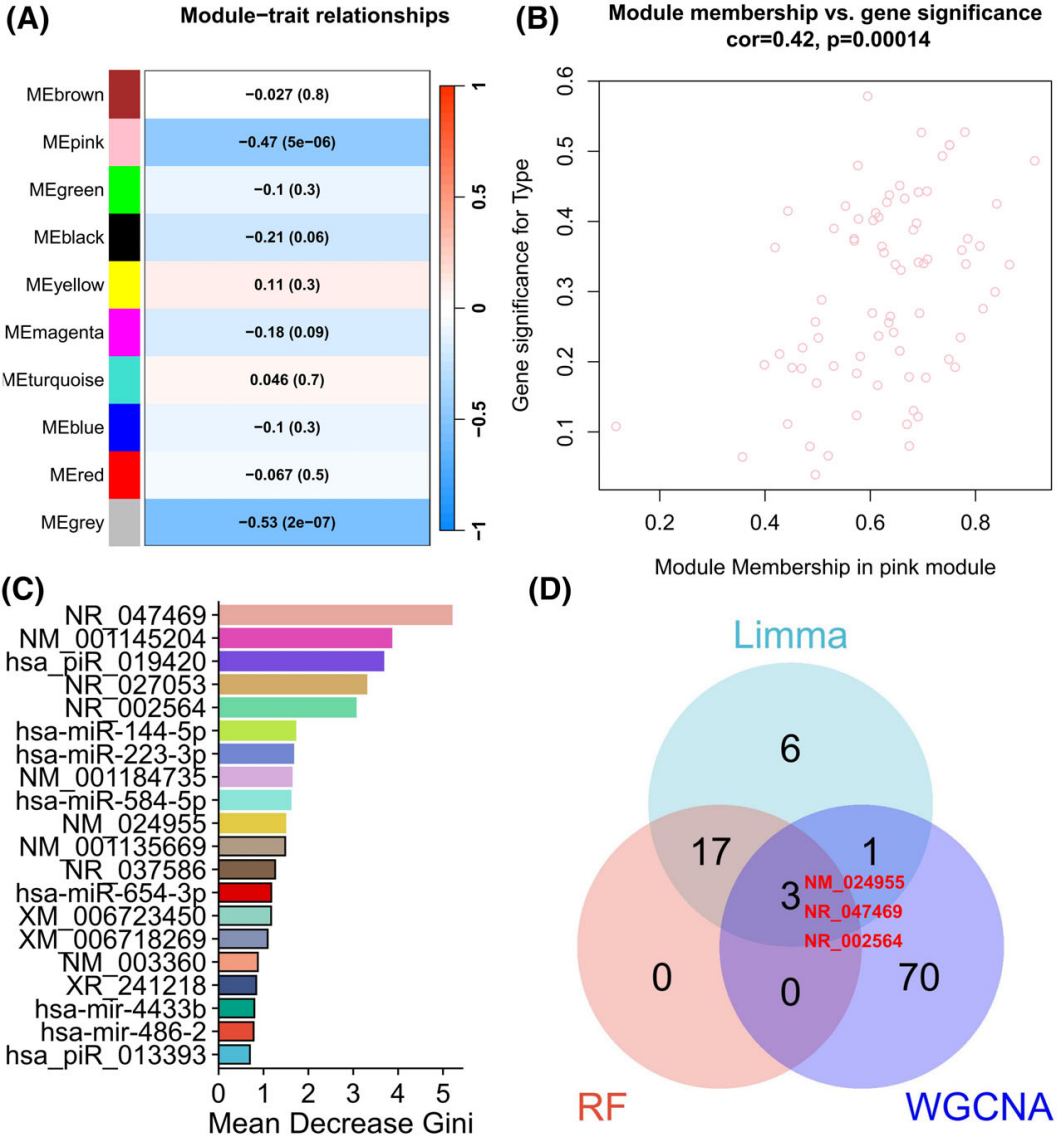
Biomarker selection of RNAs in plasma extracellular vesicles. (A–C) Weighted gene co‐expression network analysis (WGCNA) (A–B) and Random Forest (RF) (C) were used to assess the diagnostic potential of the dysregulated RNAs identified in plasma extracellular vesicles for the detection of PCa. (D) Venn diagram of overlapping genes.

To identify candidate RNA biomarkers for prostate cancer (PCa), we integrated Weighted Gene Co‐expression Network Analysis (WGCNA) with machine learning‐based feature selection. WGCNA was first performed to construct RNA co‐expression networks. Sample clustering analysis showed no obvious outliers (Fig. S2A). A soft‐thresholding power of *β* = 4 (*R* ^2^ = 0.991) was selected to ensure scale‐free network topology (Fig. S2B). Based on hierarchical clustering, a total of 10 gene modules were identified and assigned distinct colors (Fig. S2C). Among these, the pink module exhibited the strongest correlation with disease status (PCa vs. HC) (*r* = 0.42, *P* = 0.00014) (Fig. 4A,B), and was therefore considered the key disease‐associated module. To further prioritize candidate biomarkers, a Random Forest model was applied to rank RNAs based on their importance in distinguishing PCa from HC samples. The top 20 most informative RNAs were selected according to the mean decrease in Gini index (Fig. 4C). Finally, candidate biomarkers were identified by integrating results from differential expression analysis (limma), WGCNA, and Random Forest feature selection. Venn diagram analysis revealed three overlapping RNAs—NM_024955, NR_047469, and NR_002564—that were consistently identified across all three methods (Fig. 4D). These RNAs were therefore selected as key candidate biomarkers for subsequent validation.

### Validation of candidate RNA biomarkers by RT‐qPCR

**Fig. 5:**
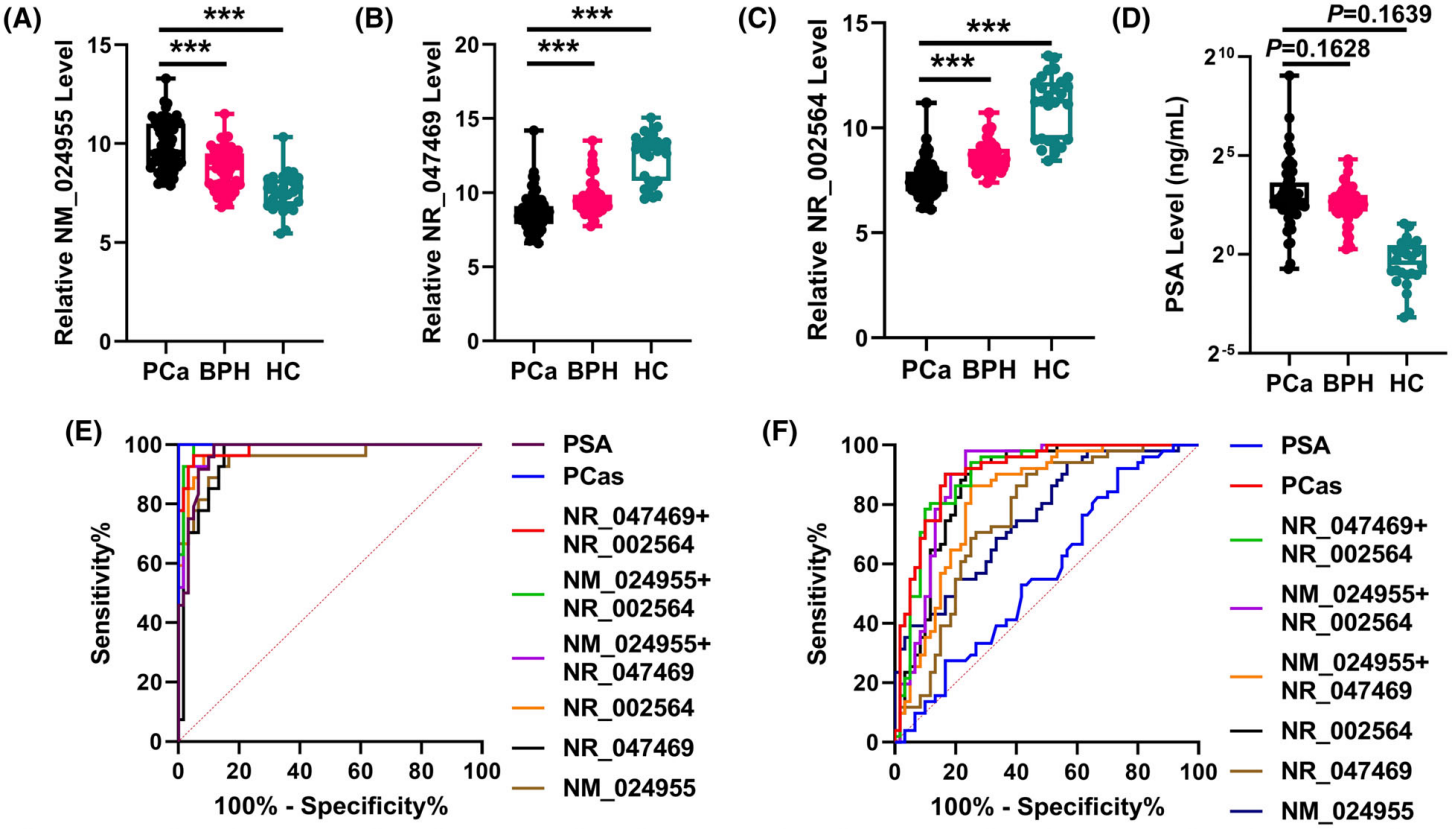
RNAs contained in plasma extracellular vesicles used as markers for diagnosing PCa. (A–D) NM_024955 (A), NR_047469 (B), NR_002564 (C), and PSA (D) in plasma extracellular vesicles from PCa patients, HCs, and BPH. Box‐and‐whisker plots show the median, interquartile range (box), and minimum‐to‐maximum values (whiskers); individual dots represent individual samples. *P*‐values were calculated using one‐way ANOVA followed by Tukey's multiple‐comparison test. (E) ROC curve analysis of the combination of NM_024955, NR_047469, NR_002564 and PSA in plasma extracellular vesicles to distinguish PCa patients and HCs. (F) ROC curve analysis of the combination of NM_024955, NR_047469, NR_002564 and PSA in plasma extracellular vesicles to distinguish PCa patients and BPHs. Each dot represents an individual clinical sample. RT‐qPCR assays were performed in technical triplicate for each sample, and expression values are presented as the mean ± SD. PCa: Prostate cancer; HCs: healthy controls; BPH:benign prostatic hyperplasia; PSA: prostate‐specific antigen. PCas: the NM_024955, NR_047469, and NR_002564 were combined using a logistic regression model. ns: *P* > 0.05; *P < 0.05;**P < 0.01; ***P < 0.001.

**Fig. 6:**
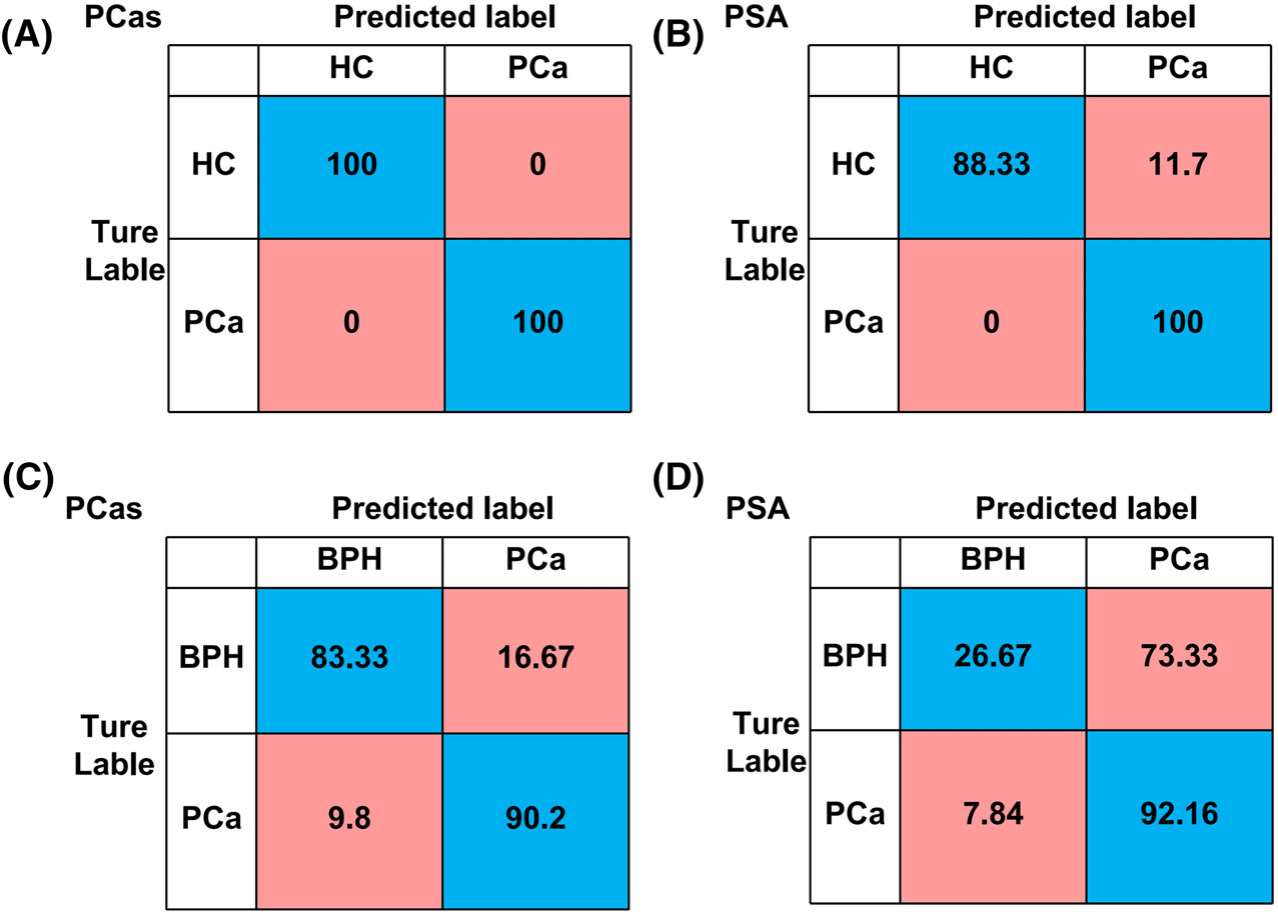
Confusion matrix of PCas and PSA in plasma extracellular vesicles to distinguish prostate cancer patients, healthy controls, and benign prostatic hyperplasia.

The expression levels of NM_024955, NR_047469, and NR_002564 were validated in an independent cohort comprising 120 patients with prostate cancer (PCa), 50 healthy controls (HCs), and 51 patients with benign prostatic hyperplasia (BPH) using RT‐qPCR. Normalization using U6 resulted in consistent expression patterns across samples. Consistent with the RNA sequencing results, NM_024955 expression was significantly elevated in PCa patients compared with both HC and BPH groups (Fig. 5A). In contrast, NR_047469 and NR_002564 were significantly downregulated in PCa samples (Fig. 5B,C). Notably, prostate‐specific antigen (PSA) levels did not show significant differences among the three groups (Fig. 5D), highlighting its limited discriminative ability in this cohort. The diagnostic performance of the three RNA biomarkers was further evaluated using receiver operating characteristic (ROC) curve analysis. Each individual RNA demonstrated the ability to distinguish PCa from both HC and BPH groups (Fig. 5E,F, Table S7). To improve diagnostic accuracy, the three RNAs were combined using a logistic regression model. The combined model achieved superior performance, with an area under the curve (AUC) of 1.0 for distinguishing PCa from HCs (100% sensitivity and specificity), and an AUC of 0.91 for differentiating PCa from BPH (90.2% sensitivity and specificity) (Fig. 5E,F, Fig. 6, Table S7). Although the combined model demonstrated excellent diagnostic performance, the relatively high AUC value should be interpreted with caution, and further validation in larger independent cohorts is required to confirm its generalizability. In comparison, PSA alone showed inferior diagnostic performance, particularly in distinguishing PCa from BPH (AUC = 0.5681), despite a relatively high AUC for PCa versus HC (AUC = 0.9719) (Fig. 5E,F, Fig. 6). Multivariate logistic regression analysis incorporating both RNA biomarkers and PSA further confirmed that the RNA panel remained an independent predictor for PCa diagnosis. Despite the differences in sample sizes between groups, consistent diagnostic trends were observed across comparisons, suggesting that the results were not solely driven by sample imbalance. These results indicate that the combined RNA biomarker panel outperforms PSA and provides improved diagnostic accuracy for PCa.

### Correlation of extracellular vesicle RNA biomarkers with clinical parameters

**Fig. 7:**
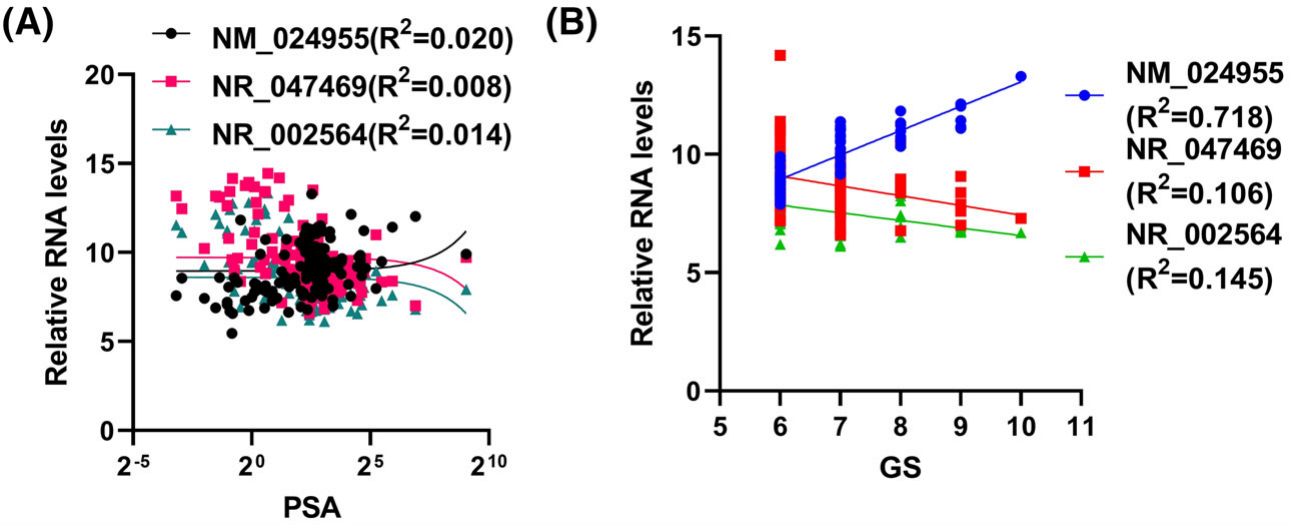
Correlation analysis of extracellular vesicle RNA biomarkers with clinical parameters. (A) Association between NM_024955, NR_047469, and NR_002564 expression levels and PSA values. (B) Relationship between NM_024955 expression and Gleason score (GS).

The associations between extracellular vesicle RNA biomarkers and clinical parameters were further evaluated. No significant correlations were observed between the expression levels of NM_024955, NR_047469, or NR_002564 and serum prostate‐specific antigen (PSA) levels (Fig. 7A). In contrast, NM_024955 expression showed a strong positive correlation with Gleason score (GS), with an *R* ^2^ value of 0.718 (Fig. 7B), suggesting a potential association with disease severity. No significant correlations with GS were observed for NR_047469 or NR_002564. These findings indicate that NM_024955 may not only serve as a diagnostic biomarker but also reflect tumor aggressiveness in PCa.

## Discussion

PCa is the second most frequently diagnosed malignancy and the fifth leading cause of cancer‐related death among men worldwide, with over 1.4 million new cases annually [1]. Although PSA testing has been widely adopted for PCa screening, its low specificity and moderate sensitivity lead to unnecessary biopsies and overtreatment, while some aggressive cases remain undetected [3, 4, 5]. PSA levels at or below 4.0 ng/mL are typically regarded as normal. Nevertheless, prostate cancer can still be present in some men whose PSA values fall under this cutoff, while many individuals with PSA readings between 4.0 and 10.0 ng/mL may not actually have the disease. This diagnostic uncertainty often compels clinicians to rely on more sophisticated and expensive techniques, such as magnetic resonance imaging, or invasive interventions such as biopsies. These additional measures not only increase patients' psychological stress but also contribute to the suboptimal allocation of medical resources.

In recent years, extracellular vesicles, particularly extracellular vesicles, have emerged as promising noninvasive biomarkers due to their stability in body fluids and their ability to carry tumor‐specific nucleic acids, proteins, and lipids reflective of the tumor microenvironment [9]. However, the diagnostic performance of extracellular vesicle RNA biomarkers for PCa remains limited by challenges in extracellular vesicle isolation and the lack of validated, clinically applicable RNA signatures. Traditional isolation techniques such as ultracentrifugation and polymer precipitation are labor‐intensive, time‐consuming, and often result in contamination with non‐vesicular material, which can compromise biomarker accuracy [10]. In this study, we performed the WGA‐conjugated magnetic bead method to isolate plasma extracellular vesicles with high specificity for glycoprotein‐rich vesicles, enabling efficient RNA recovery for transcriptomic profiling. The original alignment data for the human‐derived sequences was provided in Table S8. Through WGCNA and Random Forest modeling, we identified three extracellular vesicle RNAs—NM_024955, NR_047469, and NR_002564—that were significantly dysregulated in PCa patients compared to healthy controls and BPH cases.

NM_024955 encodes an endoplasmic reticulum (ER) resident protein known as FAD‐dependent oxidoreductase domain‐containing protein 2 (FOXRED2). FOXRED2 is a soluble, N‐glycosylated ER flavoprotein involved in endoplasmic reticulum‐associated degradation (ERAD) through interactions with ERAD components. FOXRED2 has been implicated in the pathogenesis of Alzheimer's disease (AD). Specifically, β‐amyloid protein upregulates proteasome activity primarily by inducing FOXRED2 expression, subsequently causing neuronal damage via ER stress, which may contribute to AD development [22]. Additionally, FOXRED2 mRNA is highly expressed in several human malignancies, including colorectal, breast, and pancreatic ductal adenocarcinomas. SangMi Shim et al. reported that upregulated FOXRED2 inhibits proteasome activity by disrupting 26S proteasome assembly, thereby exacerbating Aβ neurotoxicity through an ER stress response [22]. Conversely, downregulation of FOXRED2 attenuates A β‐induced cell death and ER stress [22]. FOXRED2 also reductively activates hypoxia‐targeting prodrugs such as nitro‐chloromethylbenzindolines [23], and facilitates the dislocation of specific ERAD substrates to the cytosol, suggesting a potential redox function [24]. Moreover, FOXRED2 knockdown suppresses proliferation, invasion, and migration in human melanoma cells. NR_002564 encodes the small nucleolar RNA SNORD26, a member of the C/D box snoRNA family. SNORD26 guides 2'‐O‐methylation of ribosomal RNA (rRNA), a critical posttranscriptional modification essential for ribosome biogenesis and function. By directing methylation at precise rRNA sites, SNORD26 ensures proper ribosomal assembly and structural integrity, thereby influencing protein synthesis. Emerging evidence indicates that aberrant SNORD26 expression may contribute to various diseases, including cancer, by affecting cellular growth and proliferation mechanisms. NR_047469 encodes the long intergenic non‐coding RNA (lncRNA) LINC00506, which regulates gene expression through chromatin remodeling, transcriptional, and posttranscriptional mechanisms. Recent studies have shown that LINC00506 functions as a molecular sponge for microRNAs or interacts with RNA‐binding proteins to modulate cell proliferation, migration, and apoptosis. Dysregulation of LINC00506 has been linked to cancer progression, where it promotes tumor growth and metastasis by modulating key signaling pathways. In this study, we observed a significant upregulation of NM_024955 and downregulation of SNORD26 and LINC00506 in plasma‐derived extracellular vesicles from prostate cancer patients. Previous research suggests that tumor‐derived extracellular vesicle RNAs can be transferred into immune or other tissue cells, potentially facilitating tumor immune evasion or metastasis [9]. Whether extracellular vesicle‐transported RNAs such as NM_024955 contribute to immune escape or metastatic processes in prostate cancer warrants further investigation.

The positive correlation between NM_024955 expression and Gleason score highlights its potential utility not only for diagnostic purposes but also for risk stratification in PCa. Given the well‐documented heterogeneity of PCa, there is a pressing need for biomarkers that accurately reflect disease aggressiveness to inform clinical decision‐making. The integration of NM_024955 with Gleason score offers a robust molecular tool for discriminating between indolent and aggressive disease, which could help mitigate overtreatment of low‐risk patients while facilitating timely therapeutic intervention for those with high‐risk disease.

The advantages of our approach are multifold. First, the WGA‐based capture method offers high selectivity, reproducibility, and scalability, addressing major limitations of conventional extracellular vesicle isolation methods. Second, the integration of bioinformatics and machine learning allowed for the robust selection of biomarker candidates from high‐dimensional data. Third, the combined three‐RNA panel achieved excellent diagnostic performance, with an AUC of 1.0 for PCa versus healthy controls and 0.91 for PCa versus BPH, outperforming PSA in both settings. This not only enhances diagnostic accuracy but also supports differential diagnosis, a critical clinical need.

Nevertheless, our study has limitations. The sample size was modest, and validation in larger, multicenter, and ethnically diverse cohorts is required to confirm generalizability. The cross‐sectional design limits assessment of biomarker utility for early detection or longitudinal disease monitoring. Furthermore, the molecular mechanisms through which NM_024955, NR_047469, and NR_002564 contribute to PCa biology remain speculative, warranting functional studies to elucidate their roles in tumor progression.

In conclusion, this work demonstrates that plasma extracellular vesicle RNAs identified through a lectin‐based capture and integrated bioinformatics approach can serve as highly accurate, noninvasive biomarkers for PCa diagnosis and risk assessment. The combination of innovative extracellular vesicle isolation, rigorous biomarker discovery, and strong diagnostic performance positions this study as a significant contribution toward advancing liquid biopsy technologies for PCa, with potential impact on early detection, prognosis, and personalized patient management.

### Limitations

First, although the combined biomarker model achieved excellent diagnostic performance, the relatively high AUC value may indicate potential overfitting. Future studies with larger, multicenter cohorts are required to validate the robustness and generalizability of this model. Second, U6 was used as an internal reference for RT‐qPCR normalization in this study. However, the stability of U6 in extracellular vesicles is still under debate, and it may not represent the optimal normalization control. Future studies should incorporate multiple validated reference genes or exogenous spike‐in controls to improve normalization accuracy. Third, the sample size in this study was relatively limited, and all samples were collected from a single center, which may introduce potential bias. Forth, the imbalance in sample sizes between PCa and control groups may introduce bias in statistical analyses and machine learning models. Although ROC‐based evaluation partially mitigates this issue, future studies with more balanced and larger cohorts are needed to further validate the robustness of our findings. In addition, extracellular vesicles in this study were isolated using WGA‐conjugated magnetic beads, a lectin‐based approach that captures glycosylated vesicles. Compared with antibody‐based methods, this strategy may have lower specificity and may co‐isolate nonvesicular glycosylated components. As a result, the isolated EV population may be heterogeneous, which could potentially influence the RNA profiles observed in this study. However, all samples were processed using the same isolation protocol, and EV identity was validated by TEM, NTA, and protein marker analysis, which helps ensure the reliability and comparability of the results. Future studies using orthogonal and higher‐specificity isolation methods will be important to further validate these findings. Finally, although we identified several candidate RNA biomarkers, their underlying biological mechanisms in prostate cancer progression remain largely unclear and require further functional investigation.

## Conflict of interest

The authors declare no conflict of interests.

## Author contributions

Conceptualization: WF was involved in conceptualization, resources, and writing – original draft. HW and HW were involved in investigation. HW, HW, and WF were involved in writing – review & editing.

## Supporting information


**Fig. S1.** The raw blots of western blotting for Fig. 1C.
**Fig. S2.** WGCNA analysis of plasma extracellular vesicle Transcriptomics of PCa patients and HCs. PCa: Prostate cancer; HCs: healthy controls; WGCNA: weighted gene co‐expression network analysis.

**Table S1.** Detailed demographic and clinical information for all subjects for RNA Sequencing. PCa: Prostate cancer; HCs: healthy controls; BPH: benign prostatic hyperplasia; BMI: Body Mass Index; PSA: prostate‐specific antigen; GS:Gleason score. The numbers in brackets represent variances. BMI, body mass index, calculated as weight in kilograms divided by height in meters squared. PSA, prostate‐specific antigen, measured in nanograms per milliliter (ng/mL). GS, Gleason score, calculated as the sum of the most common (primary) and second most common (secondary, ≥ 5%) histological grades (1–5) of prostate adenocarcinoma; for needle biopsy, the highest grade is used as the secondary grade if present. The score ranges from 6 to 10. Age, participant age in full years, calculated as the survey year minus the birth year, adjusted by minus one if the birthday had not yet occurred by the date of data collection. Smoker status: percentages of smokers and participants who never smoked were calculated by dividing the number of corresponding subjects by the total sample size and multiplying by 100. Alcohol consumption: The percentages of participants with alcohol intake and those who never consumed alcohol were calculated as the number of subjects in each category divided by the total sample size, multiplied by 100.

**Table S2.** Detailed demographic and clinical information for all subjects for RT‐qPCR. PCa: Prostate cancer; HCs: healthy controls; BPH:benign prostatic hyperplasia; BMI:Body Mass Index; PSA: prostate‐specific antigen; GS:Gleason score. The numbers in brackets represent variances. BMI, body mass index, calculated as weight in kilograms divided by height in meters squared. PSA, prostate‐specific antigen, measured in nanograms per milliliter (ng/mL). GS, Gleason score, calculated as the sum of the most common (primary) and second most common (secondary, ≥ 5%) histological grades (1–5) of prostate adenocarcinoma; for needle biopsy, the highest grade is used as the secondary grade if present. The score ranges from 6 to 10. Age, participant age in full years, calculated as the survey year minus the birth year, adjusted by minus one if the birthday had not yet occurred by the date of data collection. Smoker status: percentages of smokers and participants who never smoked were calculated by dividing the number of corresponding subjects by the total sample size and multiplying by 100. Alcohol consumption: The percentages of participants with alcohol intake and those who never consumed alcohol were calculated as the number of subjects in each category divided by the total sample size, multiplied by 100.

**Table S3.** Primer sequences for RT‐qPCR.

**Table S4.** Dysregulated RNAs in the plasma extracellular vesicles of prostate cancer patients and healthy controls. logFC: log_2_ fold change, calculated as log_2_(case mean expression / control mean expression); positive values indicate upregulation in prostate cancer patients, negative values indicate downregulation. AveExpr: average normalized log_2_ expression across all samples. t: moderated t‐statistic from empirical Bayes moderated *t*‐test in limma package. *P*.Value: raw *P*‐value of moderated *t*‐test. adj.P.Val: Benjamini–Hochberg adjusted *P*‐value (FDR) for multiple testing correction. B: Bayes statistic, log_2_ odds ratio that the RNA is differentially expressed between two groups; positive B values suggest higher probability of true differential expression.

**Table S5.** GO and KEGG analysis of upregulated RNAs in the plasma extracellular vesicles of prostate cancer patients. BP: Biological Process; CC: Cellular Component; MF:Molecular Function.

**Table S6.** GO and KEGG analysis of downregulated RNAs in the plasma extracellular vesicles of prostate cancer patients.

**Table S7.** ROC curve analysis. PCa: Prostate cancer; HCs: healthy controls; BPH: benign prostatic hyperplasia. AUC: Area under the receiver operating characteristic curve, ranging from 0 to 1. Values closer to 1 indicate stronger diagnostic discrimination ability; AUC > 0.5 suggests valid predictive performance, while AUC < 0.5 indicates poor single‐biomarker performance. SD: Standard deviation of AUC estimated via bootstrap resampling, representing sampling variability of the AUC value. Smaller SD indicates more stable diagnostic performance. 95%CI: 95% confidence interval of AUC. If the lower bound of the interval is greater than 0.5, the biomarker combination exhibits statistically significant discriminatory capacity.

**Table S8.** The original alignment files for the human‐derived sequences. PCa: Prostate cancer; HCs: healthy controls. Gene expression levels were quantified using RNA‐Seq by Expectation–Maximization and normalized as transcripts per million. The values presented in the table are log_2_TPM. log2TPM: log_2_‐transformed transcripts per million. TPM was calculated to normalize read counts by transcript length and total sequencing depth across samples; log_2_(TPM + 1) transformation was applied to eliminate undefined values for transcripts with zero expression. Higher log2TPM values indicate higher RNA abundance in the corresponding sample.

## Data Availability

All data generated in this study are presented within the main text and supplementary materials. Comprehensive raw datasets and supporting information can be obtained from the corresponding author following reasonable inquiry. Original alignment files for human sequences are summarized in Table S8. Restricted by internal policies of Wujin Hospital, full RNA‐seq raw data cannot be deposited for public access. Nevertheless, these RNA‐seq raw datasets may be shared solely for analytical validation after the requester executes the hospital's official non‐disclosure and data usage agreements. Queries regarding dataset details, application procedures and relevant metadata may be directed via email to weifeng09000900@163.com. All data access applications will undergo institutional internal review to verify adherence to ethical standards, patient informed consent terms and relevant regulatory requirements.
