# Supplementary material for: Accurate and noninvasive prostate cancer detection using plasma‐derived extracellular vesicle RNA
Source: FEBS Open Bio. 2026 Aug 7:10.1002/2211-5463.70305. Online ahead of print. doi: 10.1002/2211-5463.70305 (PMC13450796; doi:10.1002/2211-5463.70305)
Supplement: Supplementary file 1 [file FEB4-9999-0-s006.docx]

**Supplement data**

# Accurate and non-invasive prostate cancer detection using plasma-derived extracellular vesicle RNA

# Hanping, Wei^1,2^, Haoran, Wu^1,2^, Wei Feng^1,2,#^

Table S1. Detailed demographic and clinical information for all subjects for RNA Sequencing. PCa: Prostate cancer; HCs: healthy controls; BPH: benign prostatic hyperplasia; BMI: Body Mass Index; PSA: prostate-specific antigen; GS:Gleason score. The numbers in brackets represent variances. BMI, body mass index, calculated as weight in kilograms divided by height in meters squared. PSA, prostate-specific antigen, measured in nanograms per milliliter (ng/mL). GS, Gleason score, calculated as the sum of the most common (primary) and second most common (secondary, ≥5%) histological grades (1–5) of prostate adenocarcinoma; for needle biopsy, the highest grade is used as the secondary grade if present. The score ranges from 6 to 10. Age, participant age in full years, calculated as the survey year minus the birth year, adjusted by minus one if the birthday had not yet occurred by the date of data collection. Smoker status: percentages of smokers and participants who never smoked were calculated by dividing the number of corresponding subjects by the total sample size and multiplying by 100. Alcohol consumption: The percentages of participants with alcohol intake and those who never consumed alcohol were calculated as the number of subjects in each category divided by the total sample size, multiplied by 100.

Table S2. Detailed demographic and clinical information for all subjects for RT-qPCR. PCa: Prostate cancer; HCs: healthy controls; BPH:benign prostatic hyperplasia; BMI:Body Mass Index; PSA: prostate-specific antigen; GS:Gleason score. The numbers in brackets represent variances. BMI, body mass index, calculated as weight in kilograms divided by height in meters squared. PSA, prostate-specific antigen, measured in nanograms per milliliter (ng/mL). GS, Gleason score, calculated as the sum of the most common (primary) and second most common (secondary, ≥5%) histological grades (1–5) of prostate adenocarcinoma; for needle biopsy, the highest grade is used as the secondary grade if present. The score ranges from 6 to 10. Age, participant age in full years, calculated as the survey year minus the birth year, adjusted by minus one if the birthday had not yet occurred by the date of data collection. Smoker status: percentages of smokers and participants who never smoked were calculated by dividing the number of corresponding subjects by the total sample size and multiplying by 100. Alcohol consumption: The percentages of participants with alcohol intake and those who never consumed alcohol were calculated as the number of subjects in each category divided by the total sample size, multiplied by 100.

Table S3. Primer sequences for RT-qPCR.

Table S4. Dysregulated RNAs in the plasma extracellular vesicles of prostate cancer patients and healthy controls. logFC: log2 fold change, calculated as log₂(case mean expression / control mean expression); positive values indicate upregulation in prostate cancer patients, negative values indicate downregulation. AveExpr: average normalized log2 expression across all samples. t: moderated t-statistic from empirical Bayes moderated t-test in limma package. P.Value: raw P-value of moderated t-test. adj.P.Val: Benjamini–Hochberg adjusted P-value (FDR) for multiple testing correction. B: Bayes statistic, log2 odds ratio that the RNA is differentially expressed between two groups; positive B values suggest higher probability of true differential expression.

Table S5. GO and KEGG analysis of upregulated RNAs in the plasma extracellular vesicles of prostate cancer patients. BP: Biological Process; CC: Cellular Component; MF:Molecular Function.

Table S6. GO and KEGG analysis of downregulated RNAs in the plasma extracellular vesicles of prostate cancer patients.

Table S7. ROC curve analysis. PCa: Prostate cancer; HCs: healthy controls; BPH: benign prostatic hyperplasia. AUC: Area under the receiver operating characteristic curve, ranging from 0 to 1. Values closer to 1 indicate stronger diagnostic discrimination ability; AUC > 0.5 suggests valid predictive performance, while AUC < 0.5 indicates poor single-biomarker performance. SD: Standard deviation of AUC estimated via bootstrap resampling, representing sampling variability of the AUC value. Smaller SD indicates more stable diagnostic performance. 95%CI: 95% confidence interval of AUC. If the lower bound of the interval is greater than 0.5, the biomarker combination exhibits statistically significant discriminatory capacity.

Table S8. The original alignment files for the human-derived sequences. PCa: Prostate cancer; HCs: healthy controls. Gene expression levels were quantified using RNA-Seq by Expectation-Maximization and normalized as transcripts per million. The values presented in the table are log2TPM. log2TPM: log2-transformed transcripts per million. TPM was calculated to normalize read counts by transcript length and total sequencing depth across samples; log_2_(TPM + 1) transformation was applied to eliminate undefined values for transcripts with zero expression. Higher log2TPM values indicate higher RNA abundance in the corresponding sample.

**Figure S1. The raw blots of western blotting for Figure 1C**.

**Figure S2.** WGCNA analysis of plasma extracellular vesicle Transcriptomics of prostate cancer patients and healthy controls. PCa: Prostate cancer; HCs: healthy controls; WGCNA: weighted gene co-expression network analysis.
